# Improving pediatric emergency department physicians’ adherence to clinical practice guidelines on the diagnosis and management of group A beta-hemolytic streptococcal pharyngitis—a cross-sectional study

**DOI:** 10.1186/s12245-018-0209-4

**Published:** 2018-11-16

**Authors:** Ahmed A. Alkhazi, Khalid M. Alessa, Ahmed M. Almutairi, Hamad A. Almadi, Abdullah Akkam, Mohammed K. Almutairi, Omar A. Alhamad, Hadeel S. Ghazal

**Affiliations:** 1 Department of Pediatric, College of Medicine, Al Imam Mohammad ibn Saud Islamic University (IMSIU), Othman ibn Affan Road, Exit 5, P.O. Box: 7544, Riyadh, Saudi Arabia; 2 Pediatric Emergency Department, King Saud Medical City, Riyadh, Saudi Arabia; 3 Pediatric Emergency Department, King Abdullah Specialist Children Hospital, Riyadh, Saudi Arabia; 4 Department of Pediatric Emergency, College of Medicine, King Saud bin Abdulaziz University for Health Sciences (KSAUHS), Riyadh, Saudi Arabia

**Keywords:** Pediatric emergency medicine, Adherence to guidelines, Saudi Arabia emergency medicine, Antibiotic overuse, Centor score

## Abstract

**Introduction:**

Pharyngitis is one of the major and commonly seen presentations in pediatric emergency departments. While it could be caused by both bacterial and viral pathogens, antibiotics are improperly prescribed regardless of the pathogen. Inappropriate usage of antibiotics has risen the concern of microbial resistance and the need for stricter guidelines. Many guidelines have been validated for this reason, and the Centor score (Modified/McIsaac) is most commonly implemented. This study aims to assess the adherence and enumerate the reasons behind the suboptimal adherence to guidelines (Centor/McIsaac score) of pediatric emergency department physicians in the diagnosis and management of GABHS pharyngitis to lay the groundwork for future actions and to employ educational programs and implement local guidelines for the prevention of the development of multi-drug resistant microorganisms.

**Methodology:**

We surveyed pediatric emergency department physicians of ten teaching hospitals of Riyadh, Saudi Arabia. We used convenient sampling and estimated a sample size of 170 physicians, and interns and medical centers without pediatric emergency department were excluded from the study. Elements of the Centor score (Modified/McIsaac) were used as a part of the assessment of physicians’ knowledge of the guidelines. Adherence was assessed by requiring the participants to answer questions regarding their usage of diagnostic means when they suspect a bacterial cause of pharyngitis, as recommended by the guidelines.

**Results:**

A total of 243 physicians answered the questionnaire, 43 consultants (17.6%) and 200 non-consultants (82.4%). On the knowledge score, 9.1% scored 0, and the majority of both groups, 46.5%, earned a score of 1. The remainder 44.4%, earned a score of 2. Adherence to guidelines was defined as when diagnostic tests (throat culture or rapid antigen detection test) were always requested prior to prescribing antibiotics when acute bacterial pharyngitis was suspected. Only 27.3% (*n* = 67) of our sample are adherent to guidelines, whereas the majority, 72.7% (*n* = 175), are non-adherent. Several factors were assessed as reasons for lack of adherence.

**Conclusion:**

Lack of knowledge and adherence to guidelines is prevalent in our setting, with awareness, knowledge, and behavior of physicians playing as major factors behind this low adherence. Studies should aim towards the assessment of adherence towards locally developed guidelines.

## Introduction

Pharyngitis, colloquially known as “sore throat,” is a frequent complaint in the pediatric healthcare setting instigating hospital visits, with 1% to 2% constituting emergency department and outpatient hospital visits in the USA [1, 2]. While much of acute pharyngitis episodes are viral in origin, group A beta-hemolytic streptococcus (GABHS) accounts for about 37% of bacterial causes in children older than 5 years of age [3]. The isolation of GABHS is crucial in clinical practice to prevent suppurative and some non-suppurative post-infectious complications, and these include peri-tonsillar abscess, otitis media, and rheumatic fever [4–6].

Treatment with antibiotics for other common forms of acute bacterial pharyngitis other than GABHS is not warranted [7]. This offers an emphasis on the importance of the accurate diagnosis of GABHS, to avoid the gratuitous use of antibiotics for other causes of acute pharyngitis. The inappropriate prescription of antibiotics for respiratory infections in the emergency department raises the likelihood of bacterial drug resistance [8]. In Riyadh, Saudi Arabia, antibiotics represent the third most commonly prescribed drug group [9]. In one study describing antibiotics overuse in children with upper respiratory tract infections in Saudi Arabia, Alumran and his group had found that antibiotics were prescribed for 44 to 88% of patients presenting with upper respiratory tract infections (URTIs) [10]. Furthermore, the excess use of antibiotics was found to be more common in children with URTIs caused by a viral pathology [11]. Evidence concerning the usage of antibiotics among children in Saudi Arabia with URTIs is limited, placing an importance on the need to study its prevalence [10].

It is important to note that several strategies were used to decrease the use of antibiotics in children with respiratory tract infections. These include patient education and delayed prescription of antibiotics, which shows a reduction of up to half in the latter [12]. It is also worthy to mention that antibiotics are often prescribed in the presence of factors related to clinical presentation, and these include an ill-looking child, more particularly of younger age, fever, sore throat, and earache, along with other factors unrelated to signs and symptoms, and these include the lack of national or local guidelines, parental pressure, inaccurate diagnosis, and the fear of unfavorable clinical outcomes [13, 14]. Furthermore, an emphasis of guidelines has been on the limitation of the use of antibiotics for the treatment of acute pharyngitis in both adults and children [1]. One of the commonly used systems is the Centor score (Modified/McIsaac), which is designed to predict the likelihood of streptococcal pharyngitis in children, from ages of 3 to 17 years, presenting with a sore throat, mainly to guide the testing and treatment. The score is composed of several factors: absence of cough, swollen tender anterior cervical lymph nodes, fever of 38 °C or more, and tonsillar exudate, each account for 1 point. For a child with a score of 1 or less, neither a throat swab nor antibiotic therapy was recommended, while testing is recommended for score 2 or above. It has been shown with a modified Centor score of 1, the probability of GABHS pharyngitis is 14% and 55% of which scored 4 and above [15]^.^

In this study, we aim to assess adherence and enumerate the reasons behind the suboptimal adherence to guidelines (Centor/McIsaac score) of pediatric emergency department physicians in the diagnosis and management of GABHS pharyngitis to lay the groundwork for future actions and to employ educational programs and implement local guidelines for the prevention of the development of multi-drug resistant microorganisms.

## Methodology

### Study design

This study is a multi-center, cross-sectional study conducted in ten pediatric emergency departments of secondary and tertiary teaching hospitals in the city of Riyadh, Saudi Arabia, from September 2017 until April 2018. All pediatric emergency departments physicians (residents, specialists, fellows, and consultants) were invited to participate in a convenient sampling manner.

### Study setting and population

The study included secondary and tertiary teaching hospitals of the city of Riyadh. Most of the hospitals employ a system of 8 h per shift, with each shift being led by at least one consultant of pediatric emergency medicine. Other team members include interns, rotating residents of emergency medicine or general pediatrics, specialists, and fellows. In usual settings, consultants represent a smaller number of physicians. Both emergency and general pediatrics residency programs are 4 years in duration, while the pediatric emergency fellowship is 2 years. Centers that do not have a pediatric emergency department, centers with response rate of less than 80%, and medical interns rotating in pediatric emergency departments were excluded from the study. All centers have both a throat culture and rapid antigen test, results of rapid antigen test take about an hour, and throat culture results take approximately 2 days. In this study, we grouped the physicians into two groups: consultants and non-consultants. A sample size of 170 physicians was estimated. Participants were given 2 weeks to answer the questionnaire anonymously. Non-responders received a second e-mail and were given another 2 weeks to respond. The participants that did not respond after the second attempt were considered as non-responders. Centers were number-coded to keep track of the response rate. Ethical approval for the study was obtained from the ethical committee of the University of Imam Mohammed bin Saud Islamic University.

### Questionnaire

An Internet-based questionnaire was developed by an expert general pediatrician and three pediatric emergency medicine consultants. The questionnaire was thoroughly reviewed by a group of one family physician, one general pediatrician, one infectious diseases consultant, and one statistician (not included in the population and not part of the study group) and by the research team for validity, comprehensiveness, and appropriateness to collect the required information from the targeted population. The questionnaire was composed of 27 questions that included demographic data of gender, age, title of the physician, number of shifts per month, duration of each shift, an estimate of number of pharyngitis cases seen weekly, and decision sharing with seniors regarding case management.

After agreeing to participate in the study, the responders received a questionnaire that consist of two parts; the first part was to assess the adherence, its prevalence, and knowledge of the guidelines and the second part was to assess the non-adherent participants and the reasons behind their failure of adherence.

Adherence was investigated by asking participants about ordering diagnostic tests when there is a high suspicion of bacterial pharyngitis as recommended by the guidelines. Adherence was defined as those who would always order diagnostic tests when suspecting bacterial infection. Adherent participants had to provide a rationale for their answers before submitting their questionnaires. Knowledge of Centor score (Modified/McIsaac) was assessed by giving two validated case scenarios, where the choices were elements of Centor score (Modified/McIsaac) and these included patient’s age, presence or absence of exudate, lymphadenopathy, fever, and cough. Participants were expected to calculate the score based on their previous knowledge and answer the questions accordingly. A knowledge score of a maximum of two points was developed to assess the knowledge properly. Answering all questions correctly gives the participant 2 points, answering one question gives the participant 1 point, and not answering any questions correctly gives the participant 0 points.

Non-adherent physicians were required to complete the second part of the questionnaire and were given questions regarding the awareness of guidelines and questions regarding the factors that are assumed to be the reasons behind the suboptimal adherence; participants can choose more than one factor, which was collected by “yes” or “no” answers.

### Data analysis

Data analysis was performed using the Statistical Package for Social Sciences (SPSS), version 22. Descriptive statistics were calculated to assess the baseline demographics and socioeconomic factors. The categorical variables are presented as frequencies and percentages. A *p* value of less than 0.05 was considered as a significant statistical difference.

## Results

Table 1 shows the sociodemographic data of our sample. Sixty-six percent of the physicians are between the ages of 25–35 years, with half of our sample being males. Saudis constitute more than half of the responders. Eighty-two percent of the studied population are non-consultants. Fifty-two percent of the responders attend 16 shifts a month, 8 h per shift. Most of them seeing an average of seven cases of acute pharyngitis a week.

**Table 1:** Sociodemographic and health center-related data

	Frequency (*n* = 243)	Percentage (%)
Age group		
25–35	161	66.1%
36–45	58	24.0%
46–55	24	9.9%
Sex		
Male	143	58.7%
Female	100	41.3%
Nationality		
Saudi	160	66.1%
Non-Saudi	83	33.9%
Physician’s title		
Consultants	43	17.6%
Non-consultants	200	82.4%
Shifts/month		
Up to 16	115	47.5%
> 16	128	52.5%
Working hours each shift		
8 h	211	86.8%
12 h	32	13.2%

Table 2 presents the knowledge scores of our physicians. A total of 9.1% (*n* = 22) of the ED physicians scored 0, while the majority, of both consultant and non-consultant groups earned a score of 1. The remainder of responders, making up 44.4% (*n* = 108), have a score of TWO.

**Table 2:** Knowledge scores

			Physician’s title		Total
			Non-consultant	Consultant	
Knowledge score	0	Count	18	4	22
		%	9.0%	9.3%	9.1%
	1	Count	93	20	113
		%	46.5%	46.5%	46.5%
	2	Count	89	19	108
		%	44.5%	44.2%	44.4%
Total		Count	200	43	243
		%	100.0%	100.0%	100.0%

**Fig. 1:**
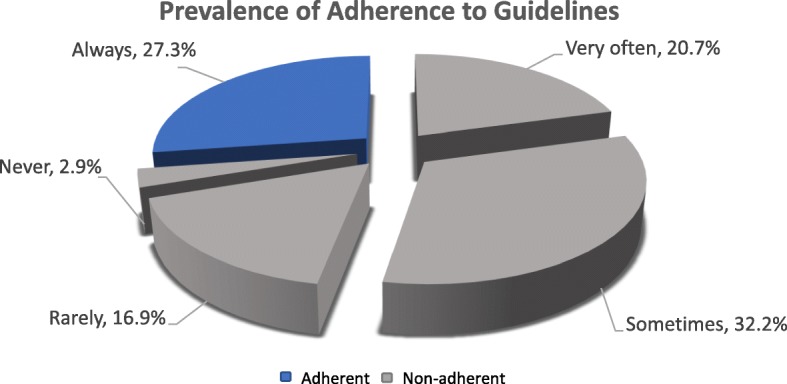
Prevalence of adherence to guidelines

Adherence to guidelines was defined as when diagnostic tests (throat culture or rapid antigen detection test) were always performed/requested prior to prescribing antibiotics when acute bacterial pharyngitis was suspected. In accordance with this, Fig. 1 demonstrates that only 27.3% (*n* = 67) are adherent to guidelines, whereas the majority, 72.7% (*n* = 175), are non-adherent.

Adherent participants were asked to rationalize always performing diagnostic tests. Guideline recommendations presented 70.6% (*n* = 47) of the answers, while hospital policy only presented 17.7% (*n* = 12). Insufficiency of signs and symptoms to reach the diagnosis represented the remaining 11.8% (*n* = 8). However, this is only for our adherent participants.

The majority of both consultants and non-consultants were non-adherent, 33 and 143 physicians, respectively. Sharing management decisions with senior physicians showed to be a statistically significant factor (*p* = 0.007), along with knowledge scores (*p* = 0.001) as elucidated by Table 3.

**Table 3:** The relationship between adherence and physician’s title (consultant and non-consultant), sharing decisions with a senior physician and knowledge scores

Variable	Adherence		*p* value
	Adherent (*n* = 67)	Non-adherent (*n* = 176)	
Title (consultant and non-consultant)			
Consultants	10	33	0.19
	15%	18.7%	
Non-consultants	57	143	
	85%	81.3%	
Sharing decisions with a senior physician			
Yes	49	99	0.007
	73.13%	56.25%	
No	18	77	
	26.9%	43.75%	
Knowledge scores			
Score	7% scored 0/2 (*n* = 5), 22% scored 1/2 (*n* = 15), 70% scored 2/2 (*n* = 47)	9% scored 0/2 (*n* = 17), 55% scored 1/2 (*n* = 98), 34% scored 2/2 (*n* = 61)	0.001

As shown in Table 4, the claim that “signs and symptoms are sufficient to make the diagnosis of acute bacterial pharyngitis” is most elected as the rationale behind not following guidelines, in both consultant and non-consultant groups, followed by difficulties in ensuring proper follow-up of patients and parental pressure to prescribe antibiotics, respectively. Unavailability of diagnostic tests is the least of all factors behind suboptimal adherence. Awareness about the clinical scoring systems that can predict GABHS is not statistically significant, in contrast to the presence of local hospital guidelines for the management of acute upper respiratory tract infections in the emergency department (*p* = 0.0004).

**Table 4:** Variables related to the main reasons behind the suboptimal adherence and awareness of guidelines of pediatric ED physicians

Factors of non-adherence	Physician’s title			*p* Value
	Consultant (*n* = 33)		Non-consultant (*n* = 143)	
Possibility for the treating physician to make the clinical diagnosis of bacterial pharyngitis with certainty without any diagnostic tests	Yes	23	131	0.489
		69.7%	91.6%	
	No	10	12	
		30.3%	8.4%	
Difficulty with collecting samples from children	Yes	12	68	0.241
		36.36%	47.55%	
	No	21	75	
		63.63%	52.44%	
Parental rejection of throat swap	Yes	6	44	0.134
		18.18%	30.8%	
	No	27	99	
		81.81%	69.2%	
Parents eventually insist on antibiotics use despite the lab results	Yes	13	77	0.133
		39.4%	53.84%	
	No	20	66	
		60.6%	46.15%	
Culture is not available at our hospital/medical center	Yes	0	20	0.27
		0%	13.98%	
	No	32	116	
		96.97%	81.11%	
	Do not know	1	7	
		3.03%	4.89%	
Rapid antigen detection test is not available at our hospital/medical center	Yes	18	61	0.45
		54.54%	42.65%	
	No	11	61	
		33.33%	42.65%	
	Do not know	4	21	
		12.12%	14.16%	
Difficulty in ensuring a proper follow-up	Yes	23	81	0.46
		69.69%	56.64	
	No	10	62	
		30.30%	43.35%	
The fear of acquiring an infection	Yes	6	39	0.29
		18.18%	27.27%	
	No	27	104	
		81.81%	72.72%	
Personal beliefs that it is not an Emergency Department procedure	Yes	12	45	0.55
		36.36%	31.46%	
	No	21	98	
		63.63%	68.53%	
Awareness about the clinical scoring systems that can predict group A streptococcal pharyngitis (e.g., Centor score (Modified/McIsaac))	Yes	22	89	0.633
		66.7%	62.2%	
	No	11	54	
		33.3%	37.8%	
Absence of local guidelines for the management of acute upper respiratory tract infections in the department	Yes	24	58	0.0004
		72.72%	40.55%	
	No	8	49	
		24.24%	34.26%	
	Do not know	1	36	
		3.03%	25.17%	

## Discussion

It is unquestionably essential to be more mindful when it comes to the prescription of antibiotics for URTIs, including pharyngitis, and this is to slow the rising rates of resistance [6]. With that in mind, physicians should be more adherent to guidelines, and not over/underprescribe antibiotics to children [16]. Utilizing the Centor score (Modified/McIsaac) when diagnosing pharyngitis improves the sensitivity and specificity when approaching these cases, and this is because viral etiologies are difficult to differentiate from bacterial etiologies clinically [17, 18].

In our study, we have found disappointing adherence rates of 27.3% of physicians. Even consultants, who should have superior knowledge and experience, are not adherent (76%). Alas, these low adherence rates have also been reported by other articles. Crocker et al. found an adherence rate of only 24% [19], and Linder et al. found that in more than half of the cases, physicians did not adhere to any guidelines in managing pharyngitis [20]. Nevertheless, our assessment of adherence did not detail any other clinical predictors that may direct physicians towards prescribing antibiotics.

Knowledge of guidelines is an important factor in adherence. Almazrou states: “Knowledge such as lack of familiarity and awareness, volume overload, time needed to stay informed and guideline accessibility are important in modifying physician practice patterns” [21]. In this study, we evaluated consultants and non-consultants’ knowledge. Surprisingly, even consultants who should have extensive knowledge of guidelines did not have a complete understanding of it, as 55.8% of consultants did not achieve a full score in the knowledge assessment, which could lead to an inappropriate practice of requesting throat cultures or prescribing antibiotics, which was identified by another study [22] to be a major contributing factor in non-adherence. However, a different study provides an opposing statement that argues that even when physicians were encouraged after educational interventions to use Centor score to differentiate between bacterial and non-bacterial pharyngitis, there was no decrease in the prescription of antibiotics [23]. It is correct to assume that knowledge influences guideline adherence, and it is also correct to assume that behavior also influences adherence, as reported by Fischer et al. [24], which we have failed to assess. Furthermore, this study was conducted in teaching hospitals; yet, most of our non-adherent group do not consult senior physicians regarding cases of pharyngitis, which could be a major problem, especially because junior physicians would treat even viral causes with antibiotics as discovered by Fakih and his group [25]. To further emphasize our point, physicians do believe resistance to antibiotics to be a definite problem but still fail to estimate the prevalence of resistance in their institutions [25], and this demonstrates how imperative it is to educate and further train physicians on the proper prescription of antibiotics.

To our knowledge, there are no articles that discussed the reasons behind suboptimal adherence in our setting. We enumerated the suspected reasons and found that “sufficiency of signs and symptoms” in the diagnosis of bacterial pharyngitis was on top of the list, which contradicts the findings of Barbosa et al. [26] and the recommendations of the Infectious Diseases Society of America (IDSA), which states that signs and symptoms are not enough [17, 18, 27]. Additionally, difficulty in ensuring proper follow-up is a major issue that physicians face when making decisions in managing cases because of the higher load on public hospitals and patients that live far away or in rural areas with little accessibility to hospitals due to the lack of public transportation systems.

Factors pertaining to patients such as parental pressure on physicians to prescribe antibiotics are important elements that should not be ignored when assessing adherence, along with other factors (e.g., beliefs that antibiotics treat viral infections, not wanting another visit) that influence physicians’ decision-making as mentioned by Alweis et al [22]. Nevertheless, according to Hedin et al., physicians who adhered to guidelines had fewer problems convincing patients not to take antibiotics, while their non-adherent counterpart had issues regarding this matter [23].

Thankfully, organizational factors of implementing guidelines were not an issue in our settings. Most of the hospitals have the diagnostic tests listed by the Centor score (Modified/McIsaac), but we have not studied this intensely. Other factors including the adoption of the guidelines by departments and evaluation of the quality were not assessed.

A review article declares that the dissemination of educational materials is a vital element of effective guideline implementation. Thus, providing educational materials (e.g., written materials, presentations, interactive conferences) is indispensable to increase awareness, understanding, and agreement with a guideline and its recommendations. Moreover, constant efforts in the training and education of health professionals are required, which may be achieved by educational assemblies, audit and feedback, workshops, and small-group collaborative training sessions [24]. Luckily, the awareness rates in both consultant and non-consultant groups of this study are high; yet, a further increase is still favorable.

To sum up, “knowledge-attitude-behavior framework” [24] dictates that physicians require awareness of guidelines and knowledge of their content. Subsequently, knowledge impacts attitude, and attitude will most definitely impact practice.

A significant number of our non-adherent group say that their institutions do not have local guidelines for the management of pharyngitis, which is not advantageous, as recommended by Fischer et al. that when guidelines are tailored to specific settings, a change in behavior could be achievable [24].

Our study implies that more efforts must be taken to change physicians’ level of knowledge by different strategies, including classes, workshops, and mandating guidelines implementation. Junior clinicians and medical students should be more involved in programs of evidence-based medicine and conducting researches that would improve the medical care system and reduce the burden of costs. Family pressure is an issue that faces those who adhere to the guidelines but could be dealt with by changing the public’s behavior towards antibiotics and influencing their awareness.

Our study had many limitations. Our data was only gathered from physician’s in the form of electronic questionnaire as it is difficult to obtain IRB from ten different hospitals; hence, access to medical files is not obtained, not to mention that some of our hospitals are still using paper files which is another obstacle. We believe that a multi-center chart review would be more helpful and authentic. Selection bias was not eradicated, given the fact that we used convenience sampling. We have not carried any interventions to investigate whether classes and discussions concerning resistance to antibiotics and utilization of guidelines increase their level of knowledge, and with this, changes to the physicians’ behaviors when it comes to guidelines. We also did not take in consideration the years of experience and undergraduate training as factors in our study.

Despite these limitations, we think our results provided a better understanding regarding the issue of not following guidelines, and the regrettable gaps in knowledge that our residents have and the importance of locally developed guidelines and how they aid in physicians’ adherence and attitude.

## Conclusion

Unfortunately, we found low rates of adherence (27.3%) for guidelines in pediatric emergency departments of teaching hospitals in Riyadh, Saudi Arabia. Both consultants and non-consultants were mostly not knowledgeable enough to achieve a full score when assessed about Centor score (Modified/McIsaac). Awareness, knowledge, and behavior of physicians are the main factors behind low adherence, emphasizing the importance of local guideline implementation and the need of employing strategies to increase physicians’ awareness and ultimately changing their behavior. Future studies should aim to implement local guidelines and assess local physicians’ adherence to evaluate whether it will improve adherence or not.

## Abbreviations

ED: Emergency department
GABHS: Group A beta-hemolytic streptococcus
URTI: Upper respiratory tract infection
